# The comparative effectiveness of mpMRI and MRI-guided biopsy vs regular biopsy in a population-based PSA testing: a modeling study

**DOI:** 10.1038/s41598-021-81459-2

**Published:** 2021-01-19

**Authors:** Abraham M. Getaneh, Eveline A. M. Heijnsdijk, Harry J. de Koning

**Affiliations:** Department of Public Health, Erasmus MC, University Medical Center Rotterdam, PO Box 2040, 3000 CA Rotterdam, The Netherlands

**Keywords:** Diseases, Oncology, Urology

## Abstract

The benefit of prostate cancer screening is counterbalanced by the risk of overdiagnosis and overtreatment. The use of a multi-parametric magnetic resonance imaging (mpMRI) test after a positive prostate-specific antigen (PSA) test followed by magnetic resonance imaging-guided biopsy (MRIGB) may reduce these harms. The aim of this study was to determine the effects of mpMRI and MRIGB vs the regular screening pathway in a population-based prostate cancer screening setting. A micro-simulation model was used to predict the effects of regular PSA screening (men with elevated PSA followed by TRUSGB) and MRI based screening (men with elevated PSA followed by mpMRI and MRIGB). We predicted reduction of overdiagnosis, harm-benefit ratio (overdiagnosis per cancer death averted), reduction in number of biopsies, detection of clinically significant cancer, prostate cancer death averted, life-years gained (LYG), and quality adjusted life years (QALYs) gained for both strategies. A univariate sensitivity analysis and threshold analysis were performed to assess uncertainty around the test sensitivity parameters used in the MRI strategy.In the MRI pathway, we predicted a 43% reduction in the risk of overdiagnosis, compared to the regular pathway. Similarly a lower harm-benefit ratio (overdiagnosis per cancer death averted) was predicted for this strategy compared to the regular screening pathway (1.0 vs 1.8 respectively). Prostate cancer mortality reduction, LY and QALYs gained were also slightly increased in the MRI pathway than the regular screening pathway. Furthermore, 30% of men with a positive PSA test could avoid a biopsy as compared to the regular screening pathway. Compared to regular PSA screening, the use of mpMRI as a triage test followed by MRIGB can substantially reduce the risk of overdiagnosis and improve the harm-benefit balance, while maximizing prostate cancer mortality reduction and QALYs gained.

## Introduction

The standard and widely used method for the detection of prostate cancer is offering transrectal ultrasound-guided biopsy (TRUSGB) for men with an elevated PSA level or abnormal digital rectal examination (DRE). However, this classical pathway is associated with an underdetection of clinically significant/high-grade prostate cancer and overdetection of clinically insignificant /low-grade prostate cancer^1^, which can lead to an unnecessary biopsy, overdiagnosis, and overtreatment. The TRUSGB is also associated with a higher rate of misclassification of grades as compared to magnetic resonance imaging-guided biopsy (MRIGB) that can lead to under or overtreatment^2^. Furthermore, TRUSGB is associated with increased risk of complications like bleeding and pain^3^, which can lead to increased health care costs and even-life threatening sepsis^4^. Therefore, looking for an alternative diagnostic pathway that can minimize the risk of overdiagnosis and maximizes the prostate cancer mortality reduction should be at urge.

Using a multi-parametric magnetic resonance imaging (mpMRI) as a triage test followed by MRIGB may reduce the risk of overdiagnosis and overtreatment. Several studies reported that the use of mpMRI and MRIGB is superior to a regular pathway^1,5,6^. The MRI pathway is characterized by having high sensitivity for clinically significant prostate cancer, and low sensitivity for insignificant cancer^7–9^, and reduces misclassification rate of grade at biopsy compared to TRUSGB^2^. Furthermore, by using this pathway, a substantial amount of unnecessary biopsies can be avoided^6^.

Although various studies reported that the use of mpMRI and MRIGB can reduce the detection of indolent prostate cancer, there is no study so far that quantifies the exact effect of this strategy on the risk of overdiagnosis as well as its effect on prostate cancer related death. However, estimation of the long-term effects of screening such as overdiagnosis is unlikely from trial data. Therefore, the aim of this modeling study was to determine the effects of mpMRI and MRIGB as compared to TRUSGB in a population-based prostate cancer screening setting.

## Materials and methods

### MISCAN model

The micro-simulation screening analysis (MISCAN) prostate cancer model^10–12^ was used to evaluate the long-term effects of prostate cancer screening using regular pathway (positive PSA test followed by TRUSGB) vs MRI pathway (positive PSA test followed by mpMRI and MRIGB). Microsimulation is a modeling technique that typically uses a large sample size of individual units (microunits), each with a unique set of attributes, and allows for simulations of downstream events on the basis of predefined states and the transition probabilities between those states over time^13^. Likewise, MISCAN prostate model is a stochastic model that simulates individual life histories, natural history of prostate cancer, effect of treatment at baseline (without screening), and the effect of screening. Each individual in the simulation starts with no prostate cancer, and through time there is a chance to transit to preclinical prostate cancer. There are eighteen pre-clinical detectable states with a combination of three stages (T1, T2, and T3), three Gleason scores (7, less than 7, and greater than 7), and two metastatic states (whether or not the cancer is metastasized). From each pre-clinical state, the tumors can progress to a more advanced state, can be clinically diagnosed, or be screen detected (Appendix_Figure S1).

After detection, the person is assigned to either radical prostatectomy (RP), radiation therapy (RT), or Active surveillance (AS). Distribution of the treatments depends on age, stage, and Gleason score as described before^14–16^. Baseline survival (in the absence of treatment) from a clinical diagnosis of prostate cancer was modeled by fitting a Cox model to Surveillance, Epidemiology, and End Results (SEER) survival data from the pre-PSA era (1983–1986), as described in a previous study^14^. The effect of treatment on survival for localized prostate cancer cases was modeled using a hazard ratio of 0.56 for those who received RP as compared with those without treatment^17^. The same effect was assumed for RT. For metastasized prostate cancer cases, it was assumed that palliative treatment has no effect on survival.

The benefit of PSA screening on prostate cancer mortality was modeled using a lead time dependent cure probability (mortality benefit increases with lead time). Lead time is the years by which detection of the cancer is advanced by screening compared with the clinical situation^16^. If a man is cured, he will not die from prostate cancer; but if he is not cured the date and cause of death are not changed due to earlier detection by screening. Death from other causes was modeled based on Dutch life table^18^.

The MISCAN prostate model was calibrated to European Randomized Study of Screening for Prostate Cancer (ERSPC) data as has been described before^12^. In order to account the younger age groups (50–54 years), the model was also calibrated to prostate cancer incidence among the Dutch population between 1989 and 2013 from age 50 to age 75 (5-years categories), and the observed prostate cancer mortality over the same period was used for validation^19^. Further descriptions about the four components of MISCAN prostate model (demography, natural history, screening and treatment) can be found at https://cisnet.flexkb.net/mp/pub/CISNET_ModelProfile_PROSTATE_ERASMUS_001_12152009_69754.pdf.

### Screening strategy

In our previous study, we compared more than 200 population-based prostate cancer screening strategies, and we found that screening with 3 years interval at ages 55–64 would be the optimum screening strategy^19^. All men with an elevated serum PSA level (cut-off 3 ng/mL) were referred to TRUSGB in that study. Those who were positive at TRUSGB were assigned to either RP, RT or AS according to the treatment distribution mentioned before. The biopsy compliance rate after a positive screen test result was assumed to be 90%, with a sensitivity of 90% as observed in the ERSPC Rotterdam data^20,21^. An 80% screening attendance rate was assumed. The total number of biopsies was calculated by using the number of screen detected cancers and a mean positive predictive value of 22.7% of a biopsy in the screen arm of the ERSPC^21^ and by using the number of clinically detected cancers and the positive predictive value of 35.8% of a biopsy in the control arm^22^.

**Figure 1:**
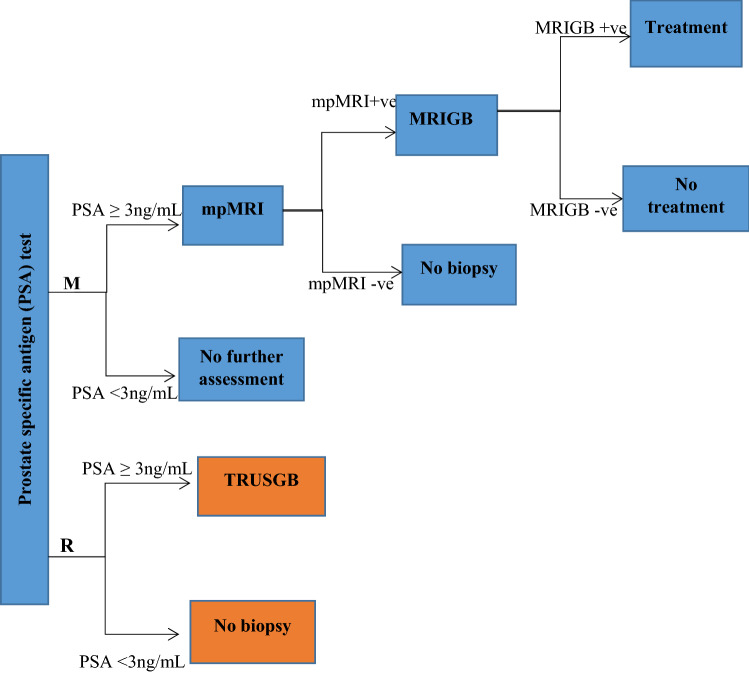
Schematic representation of the MRI pathway (M) and regular pathway (R). *mpMRI* multi parametric magnetic resonance imaging, *MRIGB* magnetic resonance imaging-guided biopsy, *MRI* magnetic resonance imaging, *TRUSGB* transrectal ultrasound-guided biopsy.

In the present study, we included mpMRI as a triage test to this screening strategy (screening with 3-year intervals at ages 55–64) for those men with an elevated PSA level (cut-off 3 ng/mL) before referring them to a biopsy (MRIGB) (Fig. 1). PIRADS scores of 3–5 were considered positive for the mpMRI test. It is important to note that we did not use a combined biopsy, rather those men with positive mpMRI tests were subjected only to an MRI-guided biopsy (no systematic biopsy). The same screening attendance and biopsy compliance were assumed as in the regular pathway. A positive predictive value of 58%^23^ was assumed to calculate the total number of biopsies in this strategy. Men positive at MRIGB were assigned to the same treatment options as in TRUSGB. Grade specific sensitivity values for mpMRI and MRIGB were mainly based on literature that used meta-analysis (Table 1). Although a very recent meta-analysis reported by Drost et al.^24^ was not included in our study, most of the test sensitivity parameter values reported in that study are within the range of the values that we used for our sensitivity analysis. We also accounted for misclassification of grades both in the MRIGB and regular biopsy. In our study misclassification of grades represents only wrong classification of clinically significant cancer in to insignificant cancer at biopsy. For the MRIGB we used an 8.7% misclassification rate based on Ahdoot et al.^2^. For the regular biopsy 16.8%, 36.3% and 60% of misclassification were reported^2^^,^^25^^,^^26^, and we used the intermediate 36.3%.

**Table 1:** Sensitivity values for the MRI pathway used in the model.

Variable	Value	References
Sensitivity of mpMRI for high grade cancer	0.94 (range 0.70–0.97)	Sathianathen et al.^37^
Overall sensitivity of mpMRI^a^	0.74 (95% CI 0.66–0.81)	de Rooij et al.^38^
Sensitivity of MRIGB for low grade cancer	0.44 (95% CI 0.26–0.64)	Schoots et al.^29^
Sensitivity of MRIGB for high grade cancer	0.91 (95% CI 0.87–0.94)	Schoots et al.^29^

*mpMRI* multi parametric magnetic resonance imaging, *MRIGB* magnetic resonance imaging-guided biopsy.

^a^Used as a sensitivity of mpMRI for low grade cancer in our model.

We compared the two strategies in terms of a harm-benefit ratio (overdiagnosis per death averted), reduction of overdiagnosis, reduction of number biopsied, detection of clinically significant cancer, death averted, life-years gained, life-years gained (LYG) per death averted, QALYs gained and QALYs gained per death averted. In this study, clinically significant prostate cancer was defined as Gleason score 7 or more and clinically insignificant cancer as Gleason score 6 and less^5^. In both screening strategies, a hypothetical cohort of 10 million men was simulated over a lifetime period. All the results are reported per 1000 men.

### Quality of life

The quality adjusted life-years (QALYs) were calculated based on the utility estimates of given health states where patients remain for a certain period of time. The utility values range between 0 (death) and 1 (perfect health), and one minus the utility value gives a loss in utility at each health state. By multiplying the number of men in a given health state with the loss in utility and the duration of the health state, the loss in quality of life was calculated. The utility estimate (0.96) and duration (1 week) for mpMRI were based on Grana et al.^27^. There is evidence that MRIGB is associated with less frequent adverse outcomes compared with TRUSGB^3^. Therefore, we assumed 50% lower disutility for MRIGB compared with that of TRUSGB. All other utilities and durations were based on our previous study^12^ (Appendix_Table S1).

### Sensitivity analysis

To check the robustness of our results, we performed a one-way sensitivity analysis on the harm-benefit ratio (overdiagnosis per death averted) of the MRI pathway. Because the performance and interpretation of both mpMRI and MRIGB are highly influenced by the specialists (radiologist or urologist) skills, we varied the test sensitivity parameters for the analysis using the 95% confidence intervals indicated in Table 1. A threshold analysis was also performed on QALYs per death averted by changing the baseline sensitivity values of the mpMRI and MRIGB simultaneously.

### Ethics declarations

No human or animal subjects are involved in this study (it is a modeling study).

## Results

### Base model

The total numbers of men referred to a biopsy were 396 and 278 for the regular and the MRI pathway respectively, a 30% reduction (Table 2). Our model predicted 16 overdiagnosed cases for the regular pathway and 9 (43% reduction) for the MRI pathway (overdiagnosed cancer was defined as a prostate cancer detected during screening but would not have been clinically diagnosed during the man’s life time in the absence of screening). The model predicted a 2.7% higher prostate cancer mortality reduction for the MRI pathway than the regular pathway (8.77 vs 8.53). The MRI based screening was also associated with a lower harm-benefit ratio (overdiagnosis per cancer death averted) than the regular screening (1.0 vs 1.8). Our model predicted a higher LY gained (85 vs 81.6) and QALYs gained (80.2 vs 77) in the MRI pathway than the regular screening pathway.

**Table 2:** Predictions of the effects of prostate cancer screening for men between age 55–64 at 3 years intervals using regular pathway and MRI pathway, per 1000 men.

	Regular pathway	MRI pathway	Difference
Number of men biopsied	396	278	118 (− 30%)
Total number of clinically insignificant^a^ cancer detected at biopsy	80.8	58.9	21.9 (− 27%)
Total number of clinically^b^ significant cancer detected at biopsy	36.0	51.3	15.3 (+ 29.8%)
Percent clinical significant cancer missed in the MRI-pathway due to reduction in biopsies	–	10.8%	–
Number overdiagnosed^c^	15.6	8.9	6.7 (− 43%)
Number of prostate cancer deaths averted	8.53	8.77	0.24 (+ 2.7%)
Overdiagnosed cases per death averted	1.8	1.0	0.8 (− 44%)
Life-years gained	81.6	85.0	3.4 (+ 4%)
LY gained per death averted	9.57	9.70	0.13 (+ 3%)
Quality adjusted life-years gained	77.0	80.2	3.2 (+ 3.9%)
QALY gained per death averted	9.0	9.14	0.14 (+ 1.5%)

^a^Clinically insignificant cancer was defined as Gleason score 6 and below (it contains both screen detected and interval cancer).

^b^Clinically significant prostate cancer was defined as Gleason score 7 or more (it contains both screen detected and interval cancer).

^c^Overdiagnosed cancer was defined as a prostate cancer detected during screening but would not have been clinically diagnosed during the man’s life time in the absence of screening.

Clinically significant prostate cancer was detected in 51.3 men in the MRI pathway, as compared with 36 in the regular pathway (30% increment in the detection rate of clinically significant prostate cancer). In contrary, fewer men were diagnosed with clinically insignificant prostate cancer in the MRI pathway than the regular pathway (59 vs 80.8), which resulted in a 27% reduction. However, the MRI pathway was also associated with an 11% risk of missing clinically significant cancer due to not performing biopsy in the mpMRI negative patients.

### Sensitivity analysis

**Figure 2:**
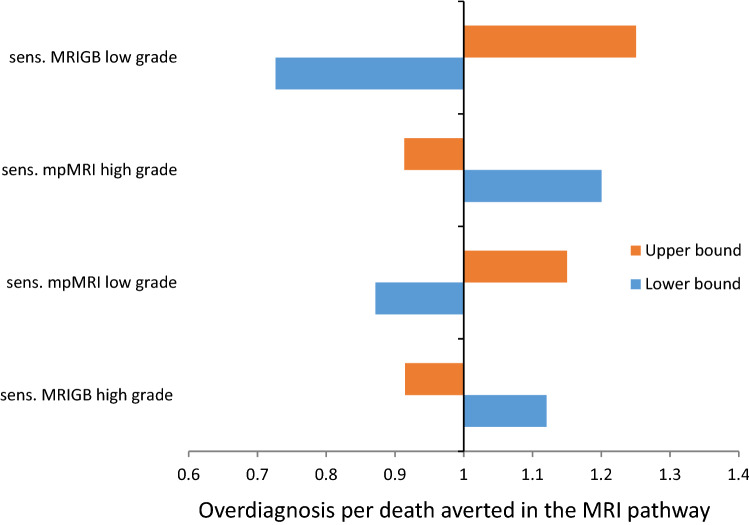
Tornedo diagram of one-way sensitivity analysis on the harm-benefit ratio (over diagnosis per cancer death averted) for the MRI pathway. sens.MRIGB low grade- sensitivity of magnetic resonance imaging-guided biopsy for low grade prostate cancer; sens.MRIGB high grade- sensitivity of magnetic resonance imaging-guided biopsy for high grade prostate cancer; sens.mpMRI low grade- sensitivity of multi-parametric magnetic resonance imaging for low grade prostate cancer; sens.mpMRI high grade- sensitivity of multi-parametric magnetic resonance imaging for high grade prostate cancer.

**Figure 3:**
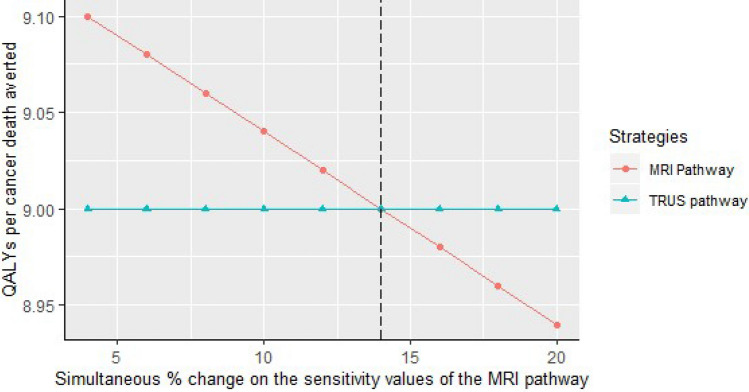
A threshold analysis diagram indicating the QALYs per cancer death averted continues to be in favor of the MRI pathway when the sensitivity values of the MRI pathway were changed simultaneously by up to 14% (this means increasing the sensitivities of mpMRI and MRIGB for low grade cancer and decreasing for high grade cancer by up to 14% simultaneously). Increasing the sensitivity for low grade cancer means detecting more Gleeson 6 cancer and decreasing the sensitivity for high grade cancer means detecting fewer clinically significant cancer which reduce the QALY per death averted.

After varying the baseline sensitivity values of the MRI pathway, using the 95% confidence intervals or ranges, the harm-benefit ratio (overdiagnosis per death averted) remained lower in the MRI pathway than the baseline value (1.8) of the regular pathway (Fig. 2). The threshold analysis indicated that when the baseline test sensitivity values of the MRI pathway were changed by 14% simultaneously, the QALYs/death averted became the same for the two strategies (Fig. 3). To be the QALYs per death averted in favour of the MRI pathway, the sensitivity of mpMRI and MRIGB for clinically significant prostate cancer should be higher than 81% and 78% respectively; whereas, for that of clinically insignificant prostate cancer it should be lower than 84% and 50% respectively.

## Discussion

The benefit of prostate cancer screening in reducing advanced stage disease or mortality is counterbalanced by the risk of overdiagnosis and overtreatment^28^. In our study, when mpMRI was applied after a positive PSA test and followed by MRIGB, the risk of overdiagnosis was decreased substantially (by 43%) compared with the regular screening. This result can be taken confirmatory for previous studies that proposed the use of mpMRI and MRIGB as a potential means to reduce the risk of overdiagnosis. The lower harm-benefit ratio predicted in the present study could also inform policymakers about the role of MRI in a population-based prostate cancer screening.

When the MRI pathway was used instead of the regular pathway, 30% of men avoided biopsies. A recent study by Kasivisvanathan et al.^5^ reported a 28% biopsy reduction due to the use of mpMRI and MRIGB. As compared to the regular pathway, the MRI pathway was also associated with a 30% higher detection rate and 27% lower detection rate for clinically significant and insignificant prostate cancer, respectively. A meta-analysis^29^ concluded that MRIGB has a higher detection rate for clinically significant prostate cancer and a lower detection rate for insignificant cancer compared with TRUSGB. More specifically, Siddiqui et al.^30^ reported MRIGB increases the detection of high risk cancer by 30% (compared to TRUSGB), and Leest et al.^31^ indicated TRUSGB would over detect insignificant cancer in 20%. The number (percentage) of clinically significant cancers reported in our study (in both pathways) are lower than the number reported by Kasivisvanathan et al. 2018, who used the same definition. The main reason for this discrepancy could be the difference in population characteristics of the two studies. For instance, the upper age limit included in the present study was 64 years, whereas in Kasivisvanathan et al. 2018 the mean age was 64 ± 7. Therefore, the older age groups in the Kasivisvanathan et al. 2018 may contribute to the higher number of high grade cancers (grade 7 and above) than reported in our study. Although it is difficult to directly compare our results with the above studies (because of differences such as, population characteristics, follow-up period and screening strategy), the general conclusion is the same: the use of mpMRI and MRI guided biopsy is superior over that of the regular pathway.

Using of the MRI pathway resulted in an increased LYG, QALYs gained, and prostate cancer death averted compared to the regular pathway. The increased in LYG and mortality benefit in the MRI pathway can be explained by the increased detection of clinically significant cancer (by about 30%), and the lower misclassification rate of grades by MRIGB (compared to TRUSGB), that were included in our model. On the other hand, the lower detection rate of clinically insignificant cancer in the MRI pathway could explain the higher QALYs gained. However, the MRI pathway also failed to detect around 11% of clinically significant cancer, that would be detected in the regular pathway, and this could explain the smaller difference in mortality benefit between the two strategies. This percentage is in agreement with a previous study by Pokorny et al.^6^. The small QALYs difference reported between the two strategies may raise a question of whether the MRI-pathway can be an efficient strategy, especially in relation to the initial additional expenditures required in the MRI-pathway. However, a substantial amount of biopsies were avoided as a result of using the MRI pathway, and this could compensate for the additional expenditures.

Our prediction of the lower harm benefit ratio (overdiagnosis per death averted) for the MRI pathway than the regular pathway was robust to the sensitivity analysis (Fig. 2). It is also important to note from the figure that, increasing the sensitivity of mpMRI and MRIGB for high grade cancer resulted in a more better harm benefit ratio, and lowering theses sensitivities relatively worse the ratio. In contrary, lowering the sensitivity of mpMRI and MRIGB for low grade cancer makes the ratio more better, and increasing these sensitivities makes the ratio relatively worse. The threshold analysis showed that when the baseline test sensitivity values of the MRI pathway were changed by 14% simultaneously (this means increasing the sensitivities of mpMRI and MRIGB for low grade cancer and decreasing for high grade cancer by 14% simultaneously), the QALYs per death averted became the same for the two strategies. This may signify the importance of adhering to proper imaging protocol as well as interpretation by the radiologist/urologist. A review by Stabile eta al^32^ indicated that there are various factors affecting the performance of mpMRI and MRIGB, among these radiologists’ reading experience and urologists’/radiologists’ biopsy experience were the main ones.

An important strength of this study is that we were able to quantify the effect of MRI based prostate cancer screening on the risk of overdiagnosis, which is obviously not observable in trial studies. We also quantified the effect of the MRI pathway on the harm-benefit ratio (overdiagnosis per death averted) as compared to the regular pathway, which was also not reported in previous studies. Furthermore, we were able to evaluate the MRI pathway in a population based screening setting. Although our model is calibrated to the Dutch prostate cancer incidence, the results may also be extrapolated to other western populations with similar prostate cancer incidence trends. Study shows that in Western Europe, the incidence of prostate cancer has been on the rise^33^.

Our study has also certain limitations. First, we assumed the same mortality benefit for radiation therapy as that of radical prostatectomy, since there is no clinical trial that compared the two treatment directly. We also assumed that treatment options will not change in both strategies. However, treatment behavior may change in the future, such as more active surveillance than now. Cost is another important factor which was not included in this study. However, avoidance of biopsies and subsequent biopsy related complications and treatment costs, probably make the MRI pathway cost-effective or at least compensate its additional costs. Various studies, though not population-based studies, indicated that the inclusion of mpMRI after a positive PSA test followed by MRI-guided biopsy is cost-effective compared to a regular prostate cancer screening pathway^34–36^. Future studies are needed to evaluate this in a population-based screening settings. Lastly, a probabilistic sensitivity analysis was not included in our study: only a one-way sensitivity analysis and threshold analysis were included.

In conclusion, our modeling results indicated that the use of mpMRI after a positive PSA test followed by MRIGB can substantially reduce the risk of overdiagnosis and improve the harm-benefit ratio, while maximizing prostate cancer mortality reduction and QALYs gained, as compared to the regular screening pathway.

## Supplementary Information

Supplementary Information
