# Supplementary material for: The comparative effectiveness of mpMRI and MRI-guided biopsy vs regular biopsy in a population-based PSA testing: a modeling study
Source: Sci Rep. 2021 Jan 19;11:1801. doi: 10.1038/s41598-021-81459-2 (PMC7815791; doi:10.1038/s41598-021-81459-2)
Supplement: Supplementary file 1 — Supplementary Information [file 41598_2021_81459_MOESM1_ESM.pdf]

The comparative effectiveness of mpMRI and MRI-guided biopsy vs regular biopsy in a population-based PSA testing: A modeling study

Abraham M. Getaneh[1], Eveline AM. Heijnsdijk[1] , and Harry J. de Koning[1]

[1]Erasmus MC, University Medical Center Rotterdam, Department of Public Health, Rotterdam, the Netherlands

Appendix table 1. Utility estimates and durations of the various health states, obtained from a previous study[12].

Health state	Utility estimates (range)	Duration
PSA screening attendance	0.99 (0.98-1.00)	1 week
Diagnostic phase	0.90 (0.87-0.94)	3 weeks
Diagnosis	0.80 (0.75-0.85)	1 month
Radical prostatectomy		
At 2 months after procedure	0.67 (0.56-0.90)	2 months
At > 2 months to 1 year after procedure	0.77 (0.70-0.91)	10 months
Radiation therapy		
At 2 months after procedure	0.73 (0.71-0.91)	
At > 2 months to 1 year after procedure	0.78 (0.61-0.88)	
Active surveillance	0.97 (0.85-1)	7 years
Postrecovery period	0.95 (0.93-1.00)	9 years
Palliative therapy	0.60 (0.24-0.86)	30 months
Terminal illness	0.40 (0.24-0.56)	6 months

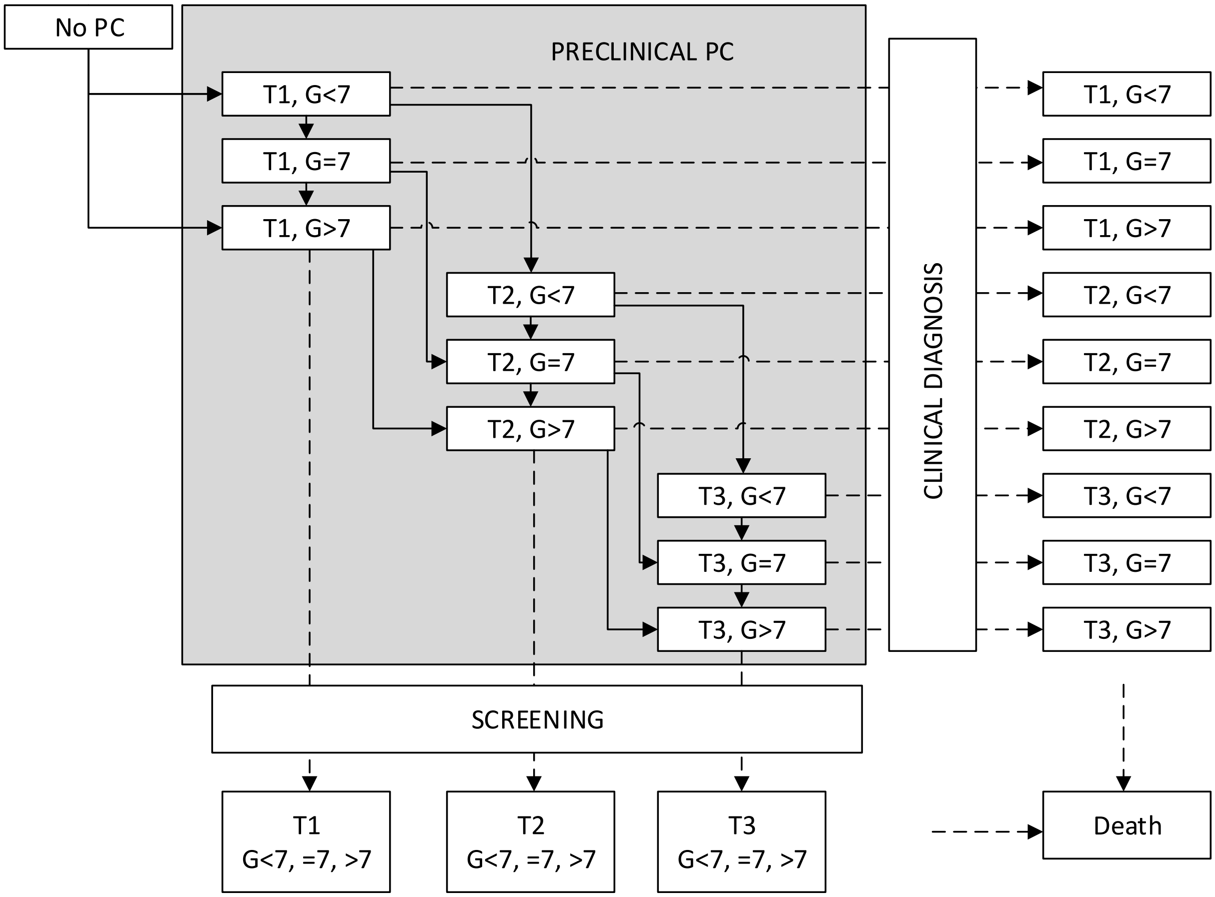

Appendix Figure 1.The MISCAN prostate cancer model. The model also contains a distinction between local and distant states, but for the sake of simplicity it is not illustrated here.

T- tumor stage ; G - Gleason score
